# Circular RNAs Variously Participate in Coronary Atherogenesis

**DOI:** 10.3390/cimb45080422

**Published:** 2023-08-13

**Authors:** Liudmila V. Dergunova, Margarita A. Vinogradina, Ivan B. Filippenkov, Svetlana A. Limborska, Alexander D. Dergunov

**Affiliations:** 1 Laboratory of Human Molecular Genetics, National Research Center “Kurchatov Institute”, Kurchatov Sq. 2, Moscow 123182, Russia; vin-rita@yandex.ru (M.A.V.); filippenkov-ib.img@yandex.ru (I.B.F.); limbor.img@yandex.ru (S.A.L.); 2 Laboratory of Structural Fundamentals of Lipoprotein Metabolism, National Medical Research Center for Therapy and Preventive Medicine, Petroverigsky Street 10, Moscow 101990, Russia; add1796@list.ru

**Keywords:** circular RNA, microRNA, coronary artery disease (CAD), biomarkers, therapy

## Abstract

Over the past decade, numerous studies have shown that circular RNAs (circRNAs) play a significant role in coronary artery atherogenesis and other cardiovascular diseases. They belong to the class of non-coding RNAs and arise as a result of non-canonical splicing of premature RNA, which results in the formation of closed single-stranded circRNA molecules that lack 5′-end caps and 3′-end poly(A) tails. circRNAs have broad post-transcriptional regulatory activity. Acting as a sponge for miRNAs, circRNAs compete with mRNAs for binding to miRNAs, acting as competing endogenous RNAs. Numerous circRNAs are involved in the circRNA–miRNA–mRNA regulatory axes associated with the pathogenesis of cardiomyopathy, chronic heart failure, hypertension, atherosclerosis, and coronary artery disease. Recent studies have shown that сirc_0001445, circ_0000345, circ_0093887, сircSmoc1-2, and circ_0003423 are involved in the pathogenesis of coronary artery disease (CAD) with an atheroprotective effect, while circ_0002984, circ_0029589, circ_0124644, circ_0091822, and circ_0050486 possess a proatherogenic effect. With their high resistance to endonucleases, circRNAs are promising diagnostic biomarkers and therapeutic targets. This review aims to provide updated information on the involvement of atherogenesis-related circRNAs in the pathogenesis of CAD. We also discuss the main modern approaches to detecting and studying circRNA–miRNA–mRNA interactions, as well as the prospects for using circRNAs as biomarkers and therapeutic targets for the treatment of cardiovascular diseases.

## 1. Introduction

Coronary artery disease (CAD), caused by atherosclerotic processes, continues to be the leading cause of morbidity and mortality in the adult population in developed countries. This disease occurs as a result of chronic inflammation of the subendothelial layer of the arteries, the accumulation of lipids and fibrous elements in their walls, the formation of coronary plaques, and the narrowing of the lumen of the vessels. Atherosclerotic damage to the vascular wall is associated with disruption of the functioning of multiple genes, epigenetic modifications, and exposure to environmental factors [1,2,3].

Transcriptomic studies indicate that a large part of the human genome is transcribed into non-coding RNAs (ncRNAs), which play a significant role in the pathogenesis of cardiovascular disease (CVD) [4]. ncRNAs are involved in the regulation of transcription [5,6], splicing, translation, and post-transcriptional regulation of gene expression [7]. Based on length and structure, ncRNAs are classified into three main categories: microRNAs (miRNAs), long ncRNAs (lncRNAs), and circular RNAs (circRNAs). miRNAs are 18–25 nucleotides long. They can bind to a recognition element in the 3′-untranslated region (3′-UTR) of mRNA, disrupting mRNA or inhibiting its translation and thus negatively controlling gene expression. It is believed that miRNAs can regulate more than half of the genes encoding human proteins [8]. By inhibiting the expression of genes involved in reverse cholesterol transport in macrophages, miRNAs play a key role in the regulation of lipid disorders and atherogenesis [9,10,11,12,13,14]. Since complementarity of 6–8 bases is sufficient to form a miRNA/mRNA duplex, a single miRNA molecule can target a wide range of different mRNAs, while a single mRNA can be attacked by several miRNAs. This creates a complex regulatory RNA–RNA network [15,16].

lncRNAs with a length of more than 200 nucleotides play an essential role in the regulation of transcription and in the post-transcriptional regulation of the expression of genes involved in the pathogenesis of atherosclerosis [9,17,18,19]. Depending on the specific interaction with DNA, RNA or proteins, lncRNAs can participate in promoter activation during transcription initiation and splicing, and change the stability and translation of cytoplasmic mRNAs [20]. lncRNAs can compete with mRNA for miRNA binding. At the same time, they reduce the effect of miRNA, which suppresses the expression of target genes. Thus, lncRNAs promote an increase in the expression of these genes [21]. These lncRNAs are considered as competing endogenous RNAs (ceRNAs).

Over the past decade, numerous studies have shown that circRNAs play a significant role in the pathogenesis of CAD and other CVDs [11,22,23,24]. circRNAs arise as a result of non-canonical premature RNA (pre-mRNA) splicing, in which closed circular molecules are formed [25]. circRNAs have broad post-transcriptional regulatory activity, acting as ceRNAs [21,26]. Acting as sponges for miRNAs [27,28], they are involved in the circRNA-miRNA–mRNA regulatory axes associated with the pathogenesis of cardiomyopathy, chronic heart failure, hypertension, atherosclerosis, and CAD [29,30,31]. With their high resistance to endonucleases, circRNAs are promising diagnostic biomarkers and therapeutic targets. Circular RNAs, underestimated for a long time, are receiving great attention now, evidenced by a noticeable growth in publications on their role in the pathogenesis of various diseases. Here we review the recent data on the involvement of circRNAs in coronary atherogenesis and highlight the main modern approaches to detecting and studying circRNA–miRNA–mRNA interactions and the major efforts in the study of mechanisms and the end-effects of circRNAs’ action on CAD. The directions of future research of circRNAs, the perspectives for their application, and possible drawbacks in CAD therapy are discussed as well.

## 2. Characteristics and Functions of circRNAs

RNA molecules that form a continuous structure were first described by Diener in 1971 when studying infectious single-stranded covalently closed RNA molecules that cause spindle tuber disease of potato (PST viroid) [32]. The term “circular RNA” (circRNA) was proposed in 1976 by Sanger et al. when they characterized the structure of viroids [33]. In 1979, the presence of circRNA in the cytoplasm of eukaryotic cells was demonstrated [34]. The detected circRNAs were considered non-functional or splicing by-products; however, with the development of high-throughput sequencing and bioinformatics approaches, it was found that circRNAs are highly expressed in all eukaryotic cells and perform certain functions [35]. Recent studies have shown that circRNAs are critical regulators of cell physiology and various pathologies by modulating gene expression [29,36,37,38,39].

Most circRNAs are formed by backsplicing exons or introns of pre-mRNA genes encoding proteins. As a result, the downstream 5′ splicing site is connected to the upstream 3′ splicing site. Thus, a circular RNA molecule with a 3′–5′-phosphodiester bond is formed [40]. The absence of terminal structures increases their stability compared to linear transcripts [35]. circRNAs can be formed from exons (ecircRNAs), introns (ciRNAs), or exon-intron sequences (elciRNAs) [35,41]. ciRNAs and elciRNAs are located in the nucleus, and their main function is cis-regulation of parental gene expression [40,42,43]. Most of the known circRNAs are ecircRNAs. They are found predominantly in the cytoplasm. The functions of circRNA include regulation of transcription mediated by polymerase II (Pol II) [40], regulation of post-transcriptional gene expression as a sponge for miRNA or protein [5,28,44,45], interaction with RNA-binding proteins (RBP), the ability to regulate their availability in the cell, modulation of protein–protein interactions [46,47] and acting as a matrix for protein translation [48]. In addition to serving as miRNA sponges, circRNAs are also associated with the storage and localization of miRNAs [49].

## 3. Biogenesis and Degradation of circRNAs

**Figure 1:**
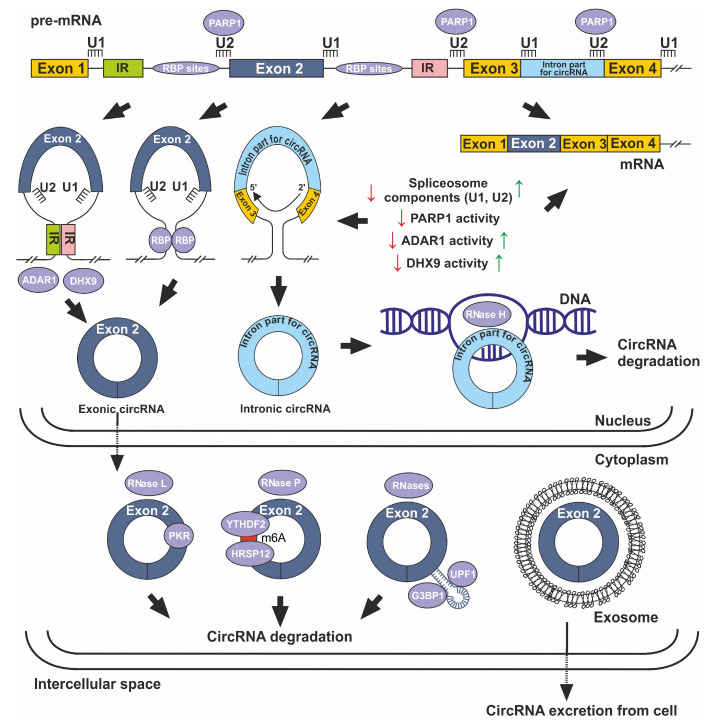
Biogenesis and degradation of circRNA. IR—inverted repeats. The green and pink colors indicate their different orientations. RBP—RNA binding protein, U1, U2—small nucleus RNAs, Red and green arrows show an increase and decrease in the amount of U1, U2, and enzyme activity, respectively.

Biogenesis and degradation of circRNA have been described in sufficient detail [25,50,51,52,53]. The pathways for circRNA formation and degradation are summarized in Figure 1. Spliceosomal machinery is regarded as a primary procedure of circRNA formation. There are three types of biogenesis model, including circularization driven by intron pairing (inverted repeat pairing) [54,55,56], circularization driven by RNA-binding protein (RBP) Muscleblind (MBL) [57], Quaking (QKI) [58], FUS [59], RBM3 [60], and others, as well as lariat-triggered circularization [35,61]. Lariats contain a significant intronic sequence and involve a 2′–5′ phosphodiester linkage at a branch point. In lariat-driven circularization, intronic lariats can be processed into circular intronic RNA (ciRNA) during splicing. The latter model is more characteristic of ciRNAs. There are also known factors that affect the accumulation of circRNAs in the cell. Among them are the restrictions on splicing and polyadelinylation factors [62], reduced activity of the ADAR1 (which reduces pairing of lateral introns) [63], and DHX9 (which unravels paired introns) enzymes [64], and the inhibition of poly (ADP-ribose) polymerase 1 (PARP1) activity [65]. Furthermore, this regulation of PARP1 within host genes acts to fine tune their transcriptional output, with implications in gene function.

Due to the stability of circRNAs compared to linear RNAs, circRNAs are capable of accumulating in cells. However, ways to reduce their numbers are also known [53]. They include RNase H-dependent cleavage of circRNAs in the nucleus when circRNAs interact with single-strand DNA during the formation of R-loops [66]. The pathways of circRNA cleavage in the cytoplasm are also shown. One of them is related to the activity of RNase L, which functions at the stage of the primary immune response to cleave viral and some cellular RNAs [67,68]. circRNAs tend to form RNA duplexes of 16–26 bp and act as endogenous inhibitors of double-stranded RNA-dependent protein kinase (PKR). Thus, it has been suggested that RNase L-mediated circRNA degradation is required for PKR activation during viral infection [68]. Another circRNA degradation pathway involves the cleavage of m6A-containing RNAs via the YTHDF2–HRSP12–RNase–P/MRP pathway [69]. Structure-mediated RNA decay (SRD) has also been described under the influence of endoribonucleases, the signal for which is the binding of UPF1 and G3BP1 proteins to highly structured circRNA motifs [70]. In the pioneering study by Hansen et al., the circRNA degradation pathway was described in an AGO-dependent manner when Ago2 cleaved circRNA after recognizing the microRNA complex [71]. It was noted that the mechanism implemented in the nucleus is apparently not common [72,73]. The separate pathway for the removal of circRNA from cells due to packaging and transportation in exosomes has been described [74,75]. The exosomes are membrane-bound extracellular vesicles (EVs) that are produced in the endosomal compartment of most eukaryotic cells [76]. EVs are nano-sized (30–150 nm) biovesicles containing DNA, RNA, and proteins. EVs are released into the surrounding extracellular fluid upon fusion between multivesicular bodies and the plasma membrane [77]. The level of circRNAs in a cell is determined by the ratio of biogenesis and degradation rates.

## 4. Main Approaches for Studying the Role of circRNA in the Pathogenesis of CAD

**Figure 2:**
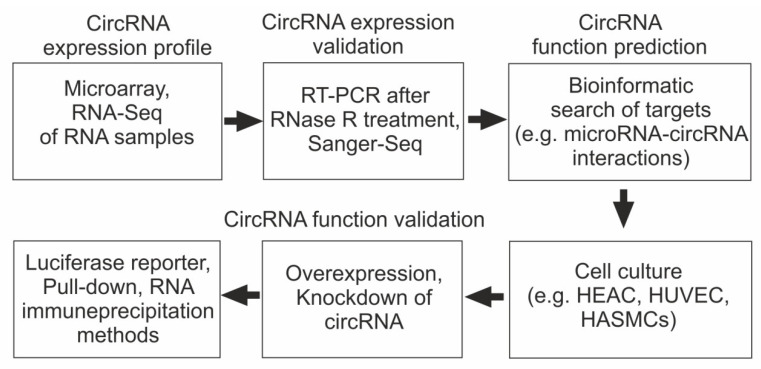
Outline of approaches used to reveal the role of circRNAs in the pathogenesis of CAD.

High-throughput RNA sequencing (RNA-Seq) has made it possible to identify thousands of circRNAs involved in physiological processes and in the pathogenesis of various diseases [78,79,80,81,82]. The most commonly used way to detect differentially expressed circRNAs (DECs) includes a comparative analysis of their representation in experimental and control biological samples using microarrays or RNA-Seq. Sanger sequencing (Sanger-Seq) and exonuclease R processing are used to confirm the cyclic structure of the molecule. The expression level of DECs in biological samples is determined using the reverse transcription polymerase chain reaction (RT-PCR) method. Bioinformatics analysis is being applied to elucidate the specific mechanisms by which circRNAs are involved in disease development. First, the search for microRNA targets of circRNAs is carried out. The selected miRNAs are then searched for mRNAs to which they can bind. The next step for the correct validation of circRNA–miRNA–mRNA interactions is their experimental validation in pathological conditions or in cell culture models of atherosclerosis in vitro. Dual-luciferase reporter (DLR) analysis, RNA pull-down (RPD) assays, and RNA immunoprecipitation (RIP) are used as experimental tools. Cultures of human aortic endothelial cells (HAEC), human umbilical vein endothelial cells (HUVEC), or human aortic smooth muscle cells (HASMC) treated with oxidized low-density lipoproteins (OX-LDL) are predominantly used to simulate atherosclerotic conditions [83]. Ox-LDLs induce an inflammatory response; proliferate, migrate towards, and invade smooth muscle cells (HASMCs); block endothelial cell proliferation; and induce cell apoptosis [84,85,86]. The scheme of methods and approaches used to identify the role of circRNAs in the pathogenesis of CAD is presented in Figure 2.

## 5. The Role of circRNAs in the Pathogenesis of CAD and Atherogenesis

The nature of cellular and molecular events in atherogenesis associated with CAD is described in detail [87,88,89]. Atherosclerosis is considered a chronic inflammatory disease caused by the accumulation of lipids in the intima of the arteries and the development of inflammatory reactions. The inflammatory process in atherogenesis is mediated by chemokines, cytokines, adhesion molecules, and other factors produced by various cells, including macrophages, endothelial cells (ECs), and vascular smooth muscle cells (VSMCs) [90,91]. EC dysfunction, transformation, abnormal proliferation and migration of VSMCs; formation of foam cells; and recruitment of macrophages, T-lymphocytes, and platelets contribute to the progression of atherosclerosis [92]. In recent years, a large number of studies have identified circRNAs involved in the regulation of gene expression associated with CAD [4,93,94]. Microarray analysis of DECs in the plasma of three CAD patients found that, compared to controls, 18 circRNAs were upregulated and six were downregulated [95]. Using miRanda software, the authors showed that nine circRNAs among them could potentially bind to hsa-miR-130a-3p. The mRNA of transient receptor potential cation channel subfamily M member 3 (TRPM3) was identified as a target for hsa-miR-130a-3p. Based on these data, a circRNA–miRNA–mRNA network with nine circRNAs and one mRNA was constructed for hsa-miR-130a-3p. Upon inhibition of hsa-miR-130a-3p, the identified circRNAs promoted the expression of the *TRPM3* gene. In another study, genome-wide transcriptome analysis of circRNAs revealed 13,160 downregulated and 12,905 upregulated circRNAs in peripheral blood mononuclear cells from five CAD patients and five controls [78]. Possible target miRNAs were identified for 10 circRNAs using miRanda and TargetScan software; their mRNA targets were identified; and circRNA–miRNA–mRNA networks were constructed using Cytoscape. Whole-transcriptome profiling of circRNA expression in segments of the coronary arteries of patients with CAD was performed. circRNAs have been identified that may play an important role in the progression of human coronary atherosclerosis and may serve as a diagnostic or therapeutic target against CAD. It should be noted that the mechanism of circRNAs’ action in studied conditions remains unknown [96,97].

A notable contribution to the study of the molecular mechanism of action of circRNAs in atherosclerosis was made by experiments confirming the axes of circRNAs, miRNAs, and mRNAs in the serum of CAD patients or in cultures of HAEC, HUVEC, or VSMC cells treated with ox-LDL as models of atherosclerosis in vitro. Quantitative analysis revealed both a decrease and an increase in the levels of different circRNAs in the studied biological samples. Table 1 lists circRNAs that are downregulated in the serum of patients with atherosclerosis or in cell cultures simulating atherosclerosis. The circRNA–miRNA–mRNA axes, the methods used to validate them, the effect, and the potential applications of circRNAs are also included for each circRNA.

Downregulated circRNAs in the plasma of patients suffering from atherosclerosis have an anti-inflammatory effect, reduce oxidative stress, and reduce vascular calcification (Table 1). The atheroprotective effect of the presented circRNAs is manifested by an increase in proliferation and a decrease in apoptosis on cultures of HAEC and HUVEC endothelial cells treated with ox-LDL, while a decrease in proliferation is noted on VSMC.

It should be noted that, being sponges for different miRNAs, individual circRNAs are involved in several circRNA–miRNA–mRNA interactions. So, for circ_0000345, three axes of circRNA–miRNA–mRNA were experimentally confirmed (Table 1) [84,104,105]. The reduced content of circ_0000345 in the serum of patients with atherosclerosis and in ASMC, HAEC, and HUVEC cell cultures treated with ox-LDL was accompanied by an increase in the content of various miRNAs. At the same time, circ_0000345 acts as a sponge for miR-647 miRNA and increases PAPD5 expression with a concomitant reduction in inflammatory response, proliferation, and ASMC migration [84]. circ_0000345 was found to be able to protect in vitro HAEC cell culture from ox-LDL-induced damage via the miR-758/CCND2 axis, promoting cell viability and cell proliferation, and inhibiting cell apoptosis [104,105]. A decrease in the level of miR-129-5p was found in experiments on the overexpression of circ_0000345 in HUVEC cells. The results confirmed the competitive endogenous role of circRNA. At the same time, the level of circ_0000345 correlated with an increase in the level of Tet methylcytosine dioxygenase 2 (TET2) mRNA [105].

The protective effect of circ_0001445 is shown, the level of which is also markedly reduced in the serum of patients with CAD [114]. Cai et al. found that overexpression of hsa_circ_0001445 in ox-LDL-induced HAEC promoted cell proliferation, inhibited cell apoptosis, suppressed the inflammatory response, and suppressed the expression of TNF-α, IL-1β and IL-16 [115]. It was shown that miRNA-640 was a direct target for hsa_circ_0001445. The authors concluded that hsa_circ_0001445 had an atheroprotective effect via miRNA-640. According to Yang et al., circ_0001445 possesses binding sites for miR-208b-5p, which in turn targets ABCG1 mRNA [110]. Binding of miR-208b-5p with circ_0001445 or ABCG1 has been confirmed using a DLR, RIP, and RPD (Table 1) [115]. Being a sponge for miR-208b-5p, circ_0001445 has an atheroprotective effect by inhibiting ox-LDL-induced HUVEC inflammation, oxidative stress, apoptosis, and foam cell formation. New circRNA–miRNA–mRNA regulatory networks have recently been constructed for hsa_circ_0001445 [116]. Bioinformatics analysis revealed an association of hsa_circ_0001445 with three miRNAs (hsa-miR-507, hsa-miR-375-3p, and hsa-miR-942-5p). The interaction network of miRNAs with 18 genes in the KEGG pathway was established; however, these results have yet to be confirmed experimentally. Thus, atherogenesis-related circRNAs, whose expression level is reduced in the plasma of patients with atherosclerosis, have an atheroprotective effect under conditions of experimental atherosclerosis in vitro and can potentially be used for CAD therapy.

Table 2 lists circRNAs that are upregulated in the serum of patients with atherosclerosis or in cell culture models of atherosclerosis. The circRNA–miRNA–mRNA axes, the methods used to validate them, the effect, and the potential applications of circRNAs are also included for each circRNA.

In the vast majority of cases, an increase in the content of circRNA is accompanied by activation of inflammation, oxidative stress, abnormal proliferation and migration of VSMC, and suppression of HUVEC proliferation. An increase in the level of circRNA can lead to an increase in the expression of ICAM-1 and VCAM-1 [135], lipid metabolism disturbances, the formation of foam cells [147,148], and other manifestations of atherosclerosis. Thus, atherogenesis-related circRNAs, whose expression level is increased with an overall proatherogenic effect, can be biomarkers and targets for CAD therapy.

One of the main risk factors for the development of atherosclerosis in humans is the increased level of cholesterol in the serum [153]. Quite recently, more than a hundred circRNA–miRNA–mRNA interactions involved in atherogenesis during foam cell formation have been described [160]. It was shown that 88 circRNAs, acting as sponges for 33 miRNAs, affect the expression of *SR-A1*, *CD36*, *ACAT2*, *ABCA1*, *ABCG1*, *ADAM10*, *APOA1*, *SCARB1*. The protein products of these genes are involved in cholesterol uptake, esterification, and efflux [161]. Thus, circRNAs are involved in coronary artery atherogenesis by various mechanisms with different circRNA–miRNA–mRNA interactions leading, in turn, to atheroprotective or proatherogenic effects in CAD.

## 6. CircRNAs Are Promising Biomarkers and Therapeutic Targets for the Treatment of CAD

The increased stability of circRNAs compared to linear transcripts, their specificity, reproducibility, and difference in expression levels in normal and pathological conditions have led to their identification as a diagnostic and prognostic marker of many diseases, including CAD [81,160,162,163]. circRNAs can be isolated from biomaterials such as cerebrospinal fluid, saliva, serum, plasma, and urine, as well as from circulating cells and exosomes [164]. The results of circRNA measurement with RT-PCR or RNA-Seq can be used for early diagnosis, treatment selection, disease prognosis, and treatment control [163]. The circRNA level can serve as a potential biomarker of CAD in different samples, such as: peripheral blood (has_circ_0124644) [165]; mononuclear cells (BTBD7_hsa_circ_0000563, hsa_circ_0001879, and has_circ_0004104) [47,166]; plasma and peripheral blood leukocytes (hsa_circ_0001445) [114,116]; and extracellular vesicles (hsa_circ_0005540) [167]. As can be seen from Table 1 and Table 2, dozens of circRNAs are currently considered risk factors for the development of CAD, making them potential biomarkers and possible targets for the treatment of this pathology. To date, there is no unequivocal answer as to whether circRNAs in blood cells or in other biomaterials reflect pathological processes occurring in atherosclerosis and, in particular, in CAD. It is still a controversial question whether signatures of gene expression in the blood can serve as biomarkers of disease states [168]. In a comparison of 15 studies in which 706 differentially expressed genes were identified, only 23 genes were replicated in no more than two or three studies. The low level of replication, according to the authors, is due to genetic differences in how an individual responds to a disease. Studies with cohorts of more than 5000 people looking at the impact of common genetic variants still failed to state that disease-specific genes may belong to biomarkers. Based on the results of the study, it was concluded that in order to overcome the heterogeneity of the response to the disease, it is necessary to use significantly larger samples of the studied case-control cohorts. At the same time, it has been shown that miRNAs are less affected by genetic differences and can more accurately reflect the disease process [168]. Thus, the question of how genetic differences can affect the content of circRNAs and whether they can be biomarkers remains open. In addition, it should be taken into account that one circRNA can adsorb several miRNAs, one miRNA molecule can target a wide range of different mRNAs, and individual circRNAs can be markers of several diseases simultaneously. An interesting idea is to use the ratios of circRNA and miRNA levels as biomarkers; for example, the circR-284/miR-221 ratio has been proposed as a marker for predicting carotid artery disease and stroke [169].

The use of circRNAs as therapeutic targets in CAD has been widely discussed [24,29,170]. An attractive therapy strategy is the creation and use of artificial circRNAs in the form of miRNA sponges [171]. The advantages and disadvantages of the methodology of circRNA synthesis in vitro have been thoroughly reviewed recently [170]. The use of newly synthesized circRNAs is restricted by imperfect cyclization, the presence of extraneous fragments, their off-target effects, and the activation of the immune response. The future improvement of circRNA synthesis technologies is required to receive safe preparations. However, RNA delivery to cells and organs is a serious problem that needs to be solved for clinical applications. Most often, viral systems are used for RNA delivery. Due to its low immunogenicity, adeno-associated virus (AAV) is one of the most promising delivery systems in translational medicine. Since AAVs do not integrate DNA into the genome of host cells, this avoids unwanted off-target changes in gene expression. Currently, most animal studies studying the cardiovascular system are focused on the application of circRNA through AAV delivery systems. A limitation of the use of AAV is the presence of a large population of neutralizing antibodies against it [170].

New horizons in modern CAD therapy for successful drug delivery were opened by EVs (exosomes) [172]. Such key qualities of EVs as low immunogenicity, high physicochemical stability, the ability to penetrate tissues, and the innate ability to bind to other cells ensured their therapeutic application [173]. Recently, the therapeutic potential of exosomes derived from circRNA_0002113 lacking mesenchymal stem cells in myocardial infarction has been shown. So, the circRNA_0002113/miR-188-3p/RUNX1 axis-mediated alleviation of apoptosis serves as a novel strategy to treat myocardial ischemia/reperfusion injury [174]. circRNAs in exosomes are being tested as potential CAD treatments, but it should be noted that the use of circRNAs in exosomes is also limited by the lack of data on the functioning of the exosomes themselves (low efficiency and safety of the proposed technologies).

Besides the difficulties in circRNA’s development and the systems of safe delivery, the existence of several points of application, several mechanisms of action, and their involvement in the progression of multiple human diseases are major drawbacks in circRNA’s use for diagnostics and therapy. Indeed, one circRNA can adsorb several miRNAs, one miRNA molecule can target a wide range of different mRNAs with the involvement of corresponding protein products in the development of several pathologies, and individual circRNAs can be markers of several diseases simultaneously. Many circRNAs, in particular circMTO1, circHIPK3, сirc_0001946, circPTPRA, and circCOL1A1, are potential targets for therapy or biomarkers of CAD (Table 1 and Table 2), and play critical roles in cancer progression as well [175,176,177,178,179]. Additional comprehensive studies of the action mechanisms of individual circRNAs and the involved regulatory networks are needed.

## 7. Conclusions

The latest achievements of the numerous studies of the expression and action mechanisms of tens of atherogenesis-related circRNAs associated with CAD are discussed here. circRNAs, as microRNA sponges, can be risk factors, potential biomarkers, and therapeutic agents for coronary artery disease. Atherogenesis-related circRNAs with decreased plasma levels in patients with atherosclerosis or in vitro conditions as a model of atherosclerosis possess anti-inflammatory and atheroprotective effects, decrease oxidative stress, and may be used potentially for CAD treatment. Other circRNAs with increased expression possess a proatherogenic effect and may serve as diagnostic and therapeutic targets. Thus, the studies of circRNAs involved in atherosclerosis of coronary arteries and their participation in various circRNA–miRNA–mRNA regulatory axes contribute both to understanding CAD development and their potential use for diagnostics and therapy.

## 8. Future Directions

The practical use of circRNAs at present is largely restricted. A majority of studies evidence the role of circRNAs as microRNA sponges; however, the full set of circRNA functions remains to be answered. Recent studies have established that circRNAs regulate gene expression by associating with RNA-binding proteins [46,47,180]. Accumulating studies indicate that circRNA encode proteins or peptides [181,182,183,184,185] that challenges the general opinion on their non-coding properties. Future studies of circular RNAs may be directed toward their regulatory and translational potential and their biogenesis and degradation. Due to the involvement of a large number of circular RNAs in various physiological and pathological processes and in the pathogenesis of various diseases, the full spectrum of circRNAs circulating in the bloodstream of CAD patients, the underlying network characteristics, and the use of large cohorts are the primary requests for the selection of promissory circRNAs as biomarkers or therapeutic agents. The relationship between circRNA activity and the genomic architecture of individuals and populations has to be considered in personalizing health care. We believe the successful treatment of cardiovascular diseases by circRNA is based upon the new circRNA–miRNA–mRNA regulatory axes and fundamental principles of circRNA biology.

## Figures and Tables

**Table 1:** List of atherogenesis-related circRNAs, whose expression level is decreased.

circRNA (Host Gene)	Source *	Interactions miRNA/mRNA	Confirmation *	Effect, (Potential Application)	Reference
circ_0026218 (*CERS5*)	HUVEC	miR-338-3p/*SIRT6*	Microarray, RT-PCR, DLR, RIP	Proliferation ↑ Inflammation ↓ Oxidative stress ↓ Apoptosis ↓ (Target for therapy)	[98]
circ_0030042 (*FOXO1*)	HUVEC	miR-616-3p/*RFX7*	RT-PCR, DLR, RIP, RPD	Proliferation ↑ Apoptosis ↓ Inflammation ↓ (Target for therapy)	[99]
circHIPK3 (*HIPK3*)	Serum, VSMC, HUVEC	miR-106a-5p/*MFN2*	RT-PCR, DLR	Osteogenic and cartilage differentiation ↓ Vascular calcification ↓ (Target for therapy)	[100]
		miR-190b/*ATG7*			[101,102]
circMTO1 (*MTO1*)	Serum, VSMC	miR-182-5p/*RASA1*	RT-PCR, DLR	Apoptosis ↑ Proliferation ↓ (Target for therapy)	[103]
circ_0000345 (*RSF1*)	Serum, ASMC	miR-647/*PAPD5*	RT-PCR, DLR, RIP	Apoptosis ↑ Proliferation ↓ Inflammation ↓ (Target for therapy)	[84]
	Serum, HAEC, HUVEC	miR-758/*CCND2*, miR-129-5p/*TET2*	RT-PCR, DLR, RIP	Proliferation ↑ Apoptosis ↓ (Target for therapy)	[104,105]
circ_06206 (*SCRG1*)	Serum, HUVEC	miR-1268b/*NR4A1*	RNA-Seq, RT-PCR, DLR	Angiogenesis ↓ (Target for therapy)	[106]
circ_0093887 (*Sirt1*)	Serum, HAEC, VSMC	miR-758-3p/*BAMBI*, miR-876-3p/*CCND2*, miR-876/*SUCNRA*, miR-132/212/*SIRT1*	RT-PCR, DLR, RIP, RPD	Proliferation ↑ Apoptosis ↓ Inflammation ↓ (Target for therapy)	[107,108,109]
circ_0001445 (*SMARCA5*)	HUVEC	miR-208b-5p/*ABCG1*	RT-PCR, DLR, RIP, RPD	Proliferation ↑ Inflammation ↓ Foam cells transformation ↓ (Biomarker, Target for therapy)	[110]
circSmoc1-2 (*Smoc1*-2)	VSMC	miR-874-3p/*ADAM19*	RNA-Seq, RT-PCR, Calcium assay, RISH	Vascular calcification ↓ (Target for therapy)	[111]
circ_0107197 (*TEX14*)	Serum, VSMC	miR-6509-3p/*THAP1*	RT-PCR, DLR, RIP	Apoptosis ↑ Proliferation ↓ (Target for therapy)	[112]
circ_0003423 (*ZNF532*)	HUVEC	miR-142-3p/*SIRT3, SOD2*	RT-PCR, DLR, RIP	Proliferation ↑ Oxidative stress ↓ Apoptosis ↓ (Target for therapy)	[113]

* Human Umbilical Vein Endothelial Cell (HUVEC), Vascular Smooth Muscle Cell (VSMC), Aortic Smooth Muscle Cell (ASMC), Human Aortic Endothelial Cell (HAEC), Reverse transcription-polymerase chain reaction (RT-PCR), Dual-luciferase reporter assay (DLR), RNA immune precipitation (RIP), pull-down assay (RPD), High-throughput RNA sequencing (RNA-Seq), RNA in situ hybridization (RISH); ↑: increase; ↓: decrease.

**Table 2:** List of atherogenesis-related circRNAs, whose expression level is increased.

circRNA (Host Gene)	Source *	Interactions miRNA/mRNA	Confirmation *	Effect,(Potential Application)	Reference
circ_0002984 (*ARHGAP32*)	Serum, VSMC	miR-326-3p/*VAMP3*, miR-665/*FGF2*	RT-PCR, DLR, RIP, RPD	Inflammation↑ Proliferation ↑ (Target for therapy)	[117,118]
circ_0003645 (*C16orf62*)	HUVEC	miR-149-3p/*TRAF7*	RT-PCR, DLR, RIP	Apoptosis ↑ Proliferation ↓ (Target for therapy)	[119]
circ_0005699 (*C16orf62*)	HUVEC	miR-450b-5P/*NFKB1*	RT-PCR, DLR	Inflammation ↑ (Target for therapy)	[120]
circ_0001946 (*CDR1*)	Serum, mouse ventricles	miR-7-5p/*PARP1*	RT-PCR, DLR, target prediction in silico	Apoptosis ↑ (Target for therapy, Biomarker)	[121,122]
circ_0026218 (*CERS5*)	Serum, HUVEC	miR-188-3p/*TLR4*	RT-PCR, DLR, RIP, RPD	Apoptosis ↑ Inflammation↑ Oxidative stress ↑ Proliferation ↓ (Target for therapy, Biomarker)	[123]
circ_0029589 (*CHFR*)	Serum, HUVEC	miR-15b-5p/*GADD45G*, miR-1197/*RAB22A*, miR-370/*FOXO1*	Microarray, RT-PCR, DLR, RPD	Apoptosis ↑ Inflammation ↑ Oxidative stress ↑ proliferation ↓ (Target for therapy)	[124,125,126]
circ_0003575 (*CHMP5*)	HUVEC	miR-532-5p/*ROCK2*	RT-PCR, DLR, RIP	Apoptosis ↑ Inflammation ↑ Proliferation ↓ (Target for therapy)	[127]
circCOL1A1 (*COL1A1*)	Serum, VSMC	miR-30a-5p/*SMAD1*	RT-PCR, RISH, DLR, RPD	VSMC transformation ↑ (Target for therapy, Biomarker)	[128]
circ_0050486 (*GPI*)	THP-1	miR-1270, miR-145/*NF1A*, *MMP16*, *USP31*	RT-PCR, DLR	Inflammation ↑ Apoptosis ↑ (Target for therapy)	[129]
	Serum, HAEC	miR-182-5p/*MYD88*	RT-PCR, DLR, RIP	Proliferation ↓ (Target for therapy, Biomarker)	[130]
circ_0044073 (*GRN*)	Serum, HUVEC, HUVSMC	miR-107/*JAK1*	RT-PCR, DLR, RPD	Proliferation ↑ (Target for therapy)	[92]
circ_0057583 (*HECW2*)	Serum, CMEC	miR-942-5p/*TLR4*	RT-PCR, DLR, RPD	Apoptosis ↑ Proliferation ↓ (Target for therapy)	[31]
circ_0091822 (*IRAK1*)	Serum, HUVEC	miR-330-5p/*TRIM14*, miR-661/*RAB22A*	RT-PCR, DLR, RIP, RPD	Inflammation ↑ Apoptosis ↑ Oxidative stress ↑ Proliferation ↓ (Biomarker, Target for therapy,)	[131,132]
circ_0018146 (*ITGB1*)	Serum	miR-342-3p/*NFAM1*	Microarray, RT-PCR, DLR, RPD	Dendritic cell maturation ↓ (Target for therapy, Biomarker)	[133]
circ_0001879 (*NIPSNAP3A*)	Serum HUVEC	miR-6873-5p/*HDAC9*	RT-PCR, DLR, RIP	Inflammation ↑ Proliferation ↓ Cholesterol transport ↓ (Target for therapy)	[134]
circ_0009135 (*NPHP4*)	Serum Monocytes, EVs	miR-1231/*EGFR*	Microarray, RT-PCR, DLR, RIP, RPD	Heterogeneous adhesion ↑ (Target for therapy, Biomarker)	[135]
circ_0033596 (*PACS2*)	HUVEC	miR-217-5p/*CLIC4*	RT-PCR, DLR, RIP	Apoptosis ↑ Proliferation ↓ (Target for therapy)	[136]
circ_0008896 (*PPAPDC1A*)	VSMC	miR-633/*CDC20B*	RT-PCR, DLR, RIP	Proliferation ↑ (Target for therapy)	[137]
circPTPRA (*PTPRA*)	Serum, VSMC	miR-636/*SP1*	RT-PCR, DLR	Proliferation ↑ Apoptosis ↓ (Target for therapy, Biomarker)	[138]
circ_0002194 (*RELL1*)	HUVEC	miR-637/*PACS2*, miR-6873-3p/*MYD88*	Microarray, RT-PCR, DLR, RIP,	Apoptosis ↑ Proliferation ↓ Inflammation ↑ Oxidative stress ↑ (Target for therapy)	[139,140]
circ_0124644 (*ROBO2*)	Serum, HASMC	miR-149/*TRAF6*	Microarray, RT-PCR, DLR, RPD	Proliferation ↑ Inflammation ↑ Apoptosis ↓ (Biomarker)	[141]
	HUVEC, Serum, CMEC	miR-370-3p/*FOXO4*, miR-186-5p/*TRIM14*	RT-PCR, DLR, RIP, RPD	Inflammation ↑ Apoptosis ↑ Proliferation ↓ (Biomarker, Target for therapy)	[142,143]
circ_0001292 (*SCAP*)	Serum, THP-1	miR-221-5p/*PDE3B*	RT-PCR, DLR, RPD	Inflammation ↑ Oxidative stress ↑ Lipid accumulation ↑ (Biomarker)	[144]
circ_102541 (*SIPA1L1*)	Serum, HUVEC	miR-296-5p/*PLK1*	RT-PCR, DLR	Proliferation ↑ Apoptosis ↓ (Target for therapy, Biomarker)	[145]
circ_0004104 (*SPARC*)	Serum, HUVEC	miR-942-5p/*ROCK2*	RT-PCR, DLR, RIP, RPD	Inflammation ↑ Apoptosis ↑ Proliferation ↓ (Target for therapy, Biomarker)	[146]
circ_0007478 (*TM7SF3*)	Serum, VSMC	miR-638/*ROCK2*	RT-PCR, DLR, RIP	Proliferation ↑ (Target for therapy Biomarker)	[147,148]
	THP-1	miR-765/*EFNA3*	Microarray, RT-PCR, DLR	Foam cells transformation ↑ (Target for therapy, Biomarker)	
circ_0021155 (*TMEM41B*)	VSMC	miR-4459/*TRPM7*	RNA-Seq, RISH, RT-PCR, DLR	Proliferation ↑ VSMC transformation ↑ (Target for therapy)	[149]
circ_0072951 (*TNPO1*)	Serum, VSMC	miR-181b/*NOTCH1*	RT-PCR, DLR	Proliferation ↑ (Target for therapy, Biomarker)	[150]
circ_0010283 (*UBR4*)	Serum, VSMC	miR-107/*ROCK*, miR-370–3p/*HMGB1*, miR-133a-3p/*PAPPA*	RT-PCR, DLR, RIP, RPD	Proliferation ↑ (Target for therapy, Biomarker)	[151,152,153]
circ_0086296 (*UHRF2*)	Carotid plaque, HUVEC, aorta of atherosclerotic mice	miR-576-3p/*IFIT1,* *STAT1*	Microarray, RT-PCR, RISH, Sanger-Seq, DLR, RIP, RPD	Inflammation ↑ Lipid accumulation ↑ (Target for therapy)	[154]
circ_0003204 (*USP36*)	Serum, VSMC	miR-182-5p/*KLF5*, miR-942-5p/*HDAC9*	RT-PCR, DLR, RPD, RIP	Proliferation ↑ (Target for therapy, Biomarker)	[155,156]
circ_0090231 (*USP9X*)	Serum, HUVEC	miR-9-5p/*TXNIP*, miR-599/*CLIC4*	RT-PCR, DLR, RIP	Apoptosis ↑ Oxidative stress ↑ Inflammation ↑ Proliferation ↓ (Target for therapy, Biomarker)	[157,158]
circ_0006896 (*VIRMA*)	EVs, HUVEC	miR-1264/*DNMT1*	Microarray, RISH, RT-PCR, DLR	Plaque formation ↑ Proliferation ↑ (Target for therapy, Biomarker)	[159]

* Vascular Smooth Muscle Cell (VSMC), Human Umbilical Vein Endothelial Cell (HUVEC), Human leukemia monocytic cell line (THP-1), Human Aortic Endothelial Cell (HAEC), Human Umbilical Vein Smooth Muscle Cell (HUVSMC), Cardiac Microvascular Endothelial Cell (CMEC), Human Aortic Smooth Muscle Cell (HASMC), Extracellular vesicles (EVs), Reverse transcription-polymerase chain reaction (RT-PCR), Dual-luciferase reporter assay (DLR), RNA immune precipitation (RIP), pull-down assay (RPD), High-throughput RNA sequencing (RNA-Seq), RNA in situ hybridization (RISH); ↑: increase; ↓: decrease.

## Data Availability

Data sharing not applicable.
